# The Relationship Between Femoral and Saphenous Nerve Conduction Changes in Individuals Without Clinical Symptoms of Neuropathy

**DOI:** 10.7759/cureus.108282

**Published:** 2026-05-05

**Authors:** Amadeusz Brzykcy, Katarzyna Kaczmarek

**Affiliations:** 1 Department of Pathophysiology of Locomotor Organs, Poznan University of Medical Sciences, Poznań, POL; 2 Student Scientific Society, Poznan University of Medical Sciences, Poznań, POL

**Keywords:** body mass index, electroneurography, femoral nerve, lower-limb flexibility, nerve conduction studies, saphenous nerve, sensory nerve action potential

## Abstract

Background: The femoral and saphenous nerves are essential components of lower-limb motor and sensory function. Nerve conduction studies (NCS) provide an objective evaluation of peripheral nerve physiology and are sensitive to early pathological changes. Limited evidence exists regarding the combined assessment of femoral motor conduction and saphenous sensory conduction in relation to anthropometric factors and flexibility.

Aim: The aim of this study is to analyze the relationships between conduction parameters of the femoral and saphenous nerves and age, body mass index (BMI), and lower-limb flexibility.

Methods: Thirty healthy young adults underwent motor conduction studies of the femoral nerve and sensory studies of the saphenous nerve stimulated at the sartorius insertion region. Latencies and amplitudes were recorded. Flexibility was assessed using the passive knee-extension angle. Statistical analyses included correlation and bilateral symmetry tests.

Results: Strong bilateral symmetry was observed for femoral compound muscle action potential (CMAP) parameters and saphenous sensory responses. Flexibility was positively associated with sensory nerve action potential (SNAP) amplitude during proximal saphenous nerve stimulation near the sartorius region. Age correlated with sensory latency, and higher body mass index was associated with reduced sensory nerve action potential amplitude and prolonged sensory latency.

Conclusions: Femoral and saphenous nerve conduction parameters show high bilateral symmetry in healthy, physically active young adults. Lower-limb soft-tissue flexibility is associated with the sensory response of the saphenous nerve, whereas age and body mass index are related to selected sensory conduction parameters. These findings suggest that individual biomechanical and anthropometric factors should be considered when interpreting sensory nerve conduction results in asymptomatic adults.

## Introduction

Peripheral nerves are responsible for functional integration between the central nervous system and the musculoskeletal system. The femoral nerve is a mixed nerve arising from the L2-L4 nerve roots. It is the largest branch of the lumbar plexus and plays a crucial role in generating knee-extension force through the innervation of the quadriceps femoris muscle [1]. It is also responsible for sensory conduction in the anterior and medial surface of the thigh. Its longest sensory branch is the saphenous nerve, which provides sensory innervation to the medial region of the leg and the infrapatellar area [2-4]. Nerve conduction can be assessed using nerve conduction studies (NCS) [5]. This test allows the assessment of the condition of the nerve fibers by analyzing latency, amplitude, and conduction velocity. The motor response can be evaluated by assessing femoral nerve conduction, while the sensory potential can be assessed by evaluating the saphenous nerve [5,6]. Both of these tests constitute the basis for diagnosing neuropathies within the innervation territory of this nerve, including post-traumatic injuries and compression-related disorders [7].

In recent years, increasing attention has been paid to the influence of the biomechanical properties of soft tissues on nerve conduction. The concept of neurodynamics assumes that nerves are subject to complex interactions with surrounding tissues through processes of sliding, tension, and compression [8]. In the case of the saphenous nerve, hamstring muscle elasticity and the tension of the fascial structures of the medial thigh and lower limb may be of significant importance. Reduced elasticity may limit nerve gliding and worsen conduction conditions [9]. Another important issue is the influence of anthropometric variables on nerve conduction parameters. Although the literature contains numerous data concerning the influence of obesity or advanced age on nerve conduction, in the population of young adults, these relationships are less clear [10,11]. Body mass index (BMI) within the normal range may not affect nerve conduction to the same extent as excessive body weight, but there is a lack of clear research in this area. To date, few publications have simultaneously analyzed conduction in the femoral and saphenous nerves. The present study addresses this issue, providing normative data and analyzing relationships relevant for clinical diagnostics. The aim of the study was to analyze the relationships between conduction parameters of the femoral and saphenous nerves and age, BMI, and lower-limb flexibility.

## Materials and methods

The study was conducted at the Department of Pathophysiology of Locomotor Organs, Poznan University of Medical Sciences. The Bioethics Committee of Karol Marcinkowski University of Medical Sciences in Poznań issued approval 217/25. Data collection was performed over two months, starting in November 2025. A priori sample size was estimated using the Fisher z-transformation for correlation analysis, assuming an expected correlation coefficient of r = 0.50, two-tailed significance level α = 0.05, and statistical power (1-β) = 0.80. The minimum required sample size was calculated as n = ((Z_α/2 + Z_β) / arctanh(r))² + 3, yielding n = 30 participants. This requirement was met by the final study sample.

A total of 53 healthy volunteers initially agreed to participate in the study. Thirty-one individuals completed the preliminary neurological screening, while the remaining volunteers either did not attend the qualification visit or did not meet the inclusion criteria before screening. One screened participant was excluded after examination due to not meeting the inclusion criteria. The reasons for exclusion included the presence of stretch signs, asymmetrical reflexes, sensory asymmetry, and intolerance to the nerve conduction examination.

Ultimately, 30 healthy participants were included in the analysis. The inclusion criteria were age of 18-40 years, the absence of pain complaints, no history of injuries, no impairment of the central or peripheral nervous system, regular physical activity including at least two training sessions of high intensity per week lasting more than 40 minutes, and qualification confirmed by a single physician (neurologist). The exclusion criteria were refusal to provide informed consent; chronic pain of the spine, hip, or knee; a history of surgical procedures in the lower limb, cardiovascular disease, neurological disease, diabetes mellitus, peripheral neuropathy, epilepsy, pregnancy, active infection, or inflammatory disease; a history of stroke, oncological disease, use of electronic life-sustaining devices, coagulation disorders, or hemophilia; and the current use of anticoagulant therapy.

The neurological examination included an assessment of spinal mobility during lateral flexion, the perception of superficial sensation, deep sensation, and vibration. Stretch signs were evaluated: the femoral nerve stretch test, the crossed straight leg raise (CSLR) test, and the straight leg raise (SLR) test [12-14]. The knee and ankle reflexes were assessed, as well as muscle strength using the Lovett scale for the rectus femoris muscle and the tibialis anterior muscle, bilaterally [15]. Additionally, as part of the functional assessment, the fingertips-to-floor test and the popliteal angle measurement were performed.

The examined group consisted of 11 men and 19 women. The age of the women ranged from 20 to 35 years and that of the men from 20 to 37 years; the mean age of the entire population was 23.47 ± 4.38 years. The height of the women ranged from 1.54 to 1.74 m, whereas in men, it ranged from 1.73 to 1.94 m; the mean value for the whole group was 1.73 ± 0.09 m. Body mass in women ranged from 51 to 77 kg and in men from 61 to 94 kg, with a mean value for the entire group of 66.67 ± 12.2 kg. BMI values in the female population ranged from 17.86 to 27.94 kg/m² and in men from 19.53 to 29.66 kg/m²; the mean value for the study group was 22.11 ± 2.82 kg/m². The above anthropometric data are presented in Table 1.

**Table 1:** Anthropometric data (n = 30) BMI: body mass index

Parameter	Female (n = 19)	Male (n = 11)	Total
Age (years)	22.52 ± 3.25	25.09 ± 5.66	23.47 ± 4.38
Height (m)	1.67 ± 0.07	1.83 ± 0.06	1.73 ± 0.09
Weight (kg)	60.73 ± 8.69	76.91 ± 11.51	66.67 ± 12.20
BMI (kg/m²)	21.71 ± 3.25	22.78 ± 3.12	22.11 ± 2.82

During the functional assessment, a deficit in the fingertips-to-floor test was observed in seven participants, with a mean deficit of 11 ± 3.84 cm, while in the popliteal angle measurement, the range of motion was below the norm by 30 ± 5.7 degrees in 16 examined limbs.

NCS of the femoral nerve and the saphenous nerve were performed according to the methodology described in the textbook Atlas of Nerve Conduction Studies and Electromyography [16]. Motor and sensory nerve fiber conduction in the lower limb was analyzed by evaluating latency, amplitude, and nerve impulse conduction velocity [17].

The examinations were performed using the KeyPoint Diagnostic System (Medtronic, Copenhagen, Denmark) equipped with an electrical stimulator and an NCS signal recording module. Ag/AgCl surface electrodes with a 5 mm² active surface area were used. The room temperature was maintained at 22°C, ensuring the stability of nerve conduction parameters. The skin temperature of the examined limb was verified to be ≥36.4°C prior to each examination, in accordance with established technical standards [18]. The equipment settings included a high-pass filter of 20 Hz, a low-pass filter of 10 kHz, a gain of 100-5000 µV, a time base of 8 ms per division, and rectangular pulses with a duration of 0.2 ms and a frequency of 1 Hz. Each measurement was repeated twice to confirm recording stability [18].

**Figure 1:**
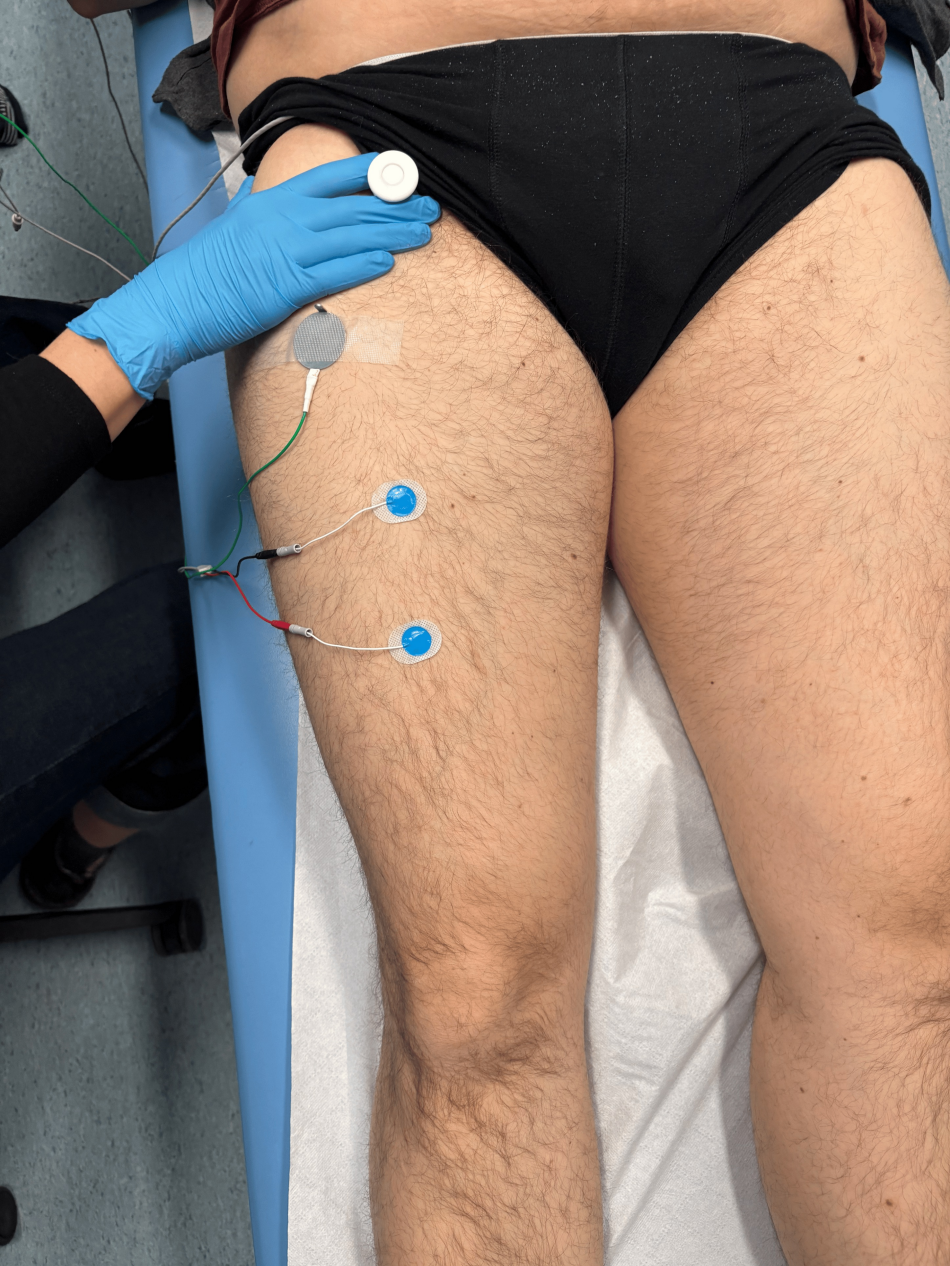
Femoral nerve stimulation

The femoral nerve examination was performed according to the following protocol: the stimulating electrode was placed medially to the palpable pulse of the femoral artery in the region of the inguinal ligament, corresponding to the anatomical course of the femoral nerve as shown in Figure 1. Rectangular pulses with a duration of 0.2 ms, a frequency of 1 Hz, and an intensity gradually increased from 0 to 80 mA were applied until the maximal muscle potential was obtained [19]. The active electrode was placed on the rectus femoris muscle, whereas the reference electrode was placed on the tendon of the rectus femoris muscle. The ground electrode was positioned midway between the stimulating and recording electrodes [16]. The following parameters were analyzed: compound muscle action potential (CMAP) amplitude in mV, distal latency in ms, and nerve conduction velocity (NCV) calculated based on the distance between the stimulation site and the recording electrode.

**Figure 2:**
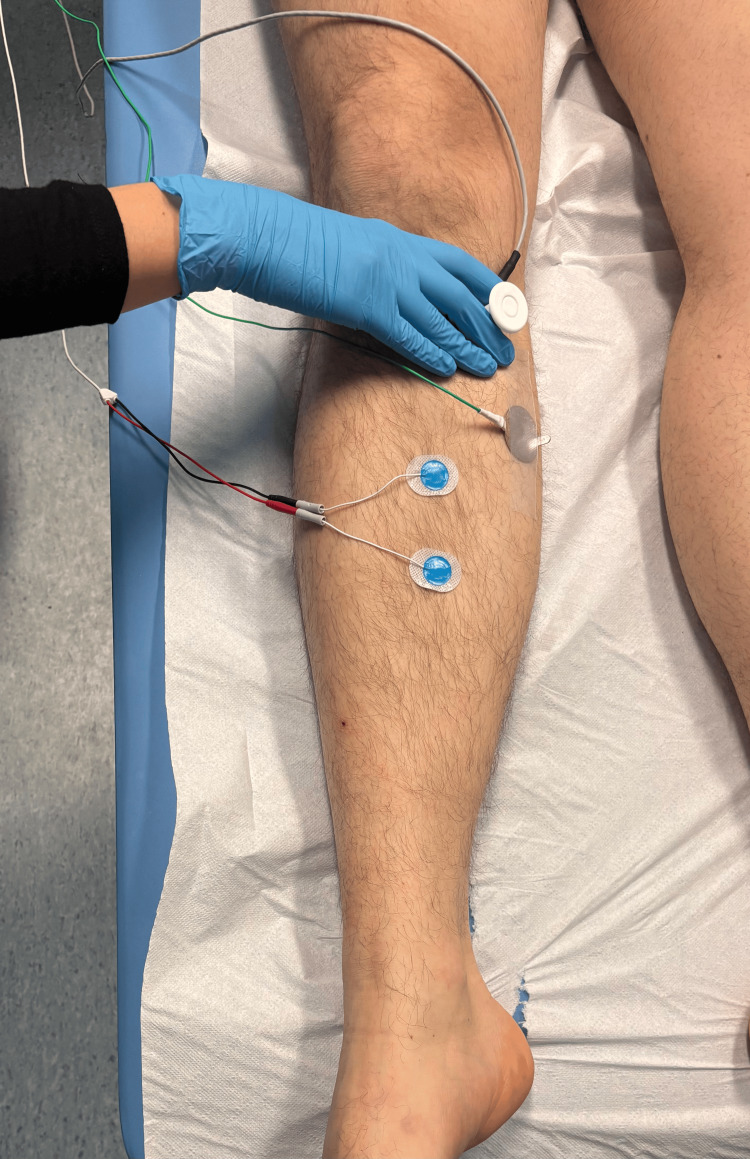
Saphenous nerve stimulation

For the saphenous nerve, the stimulating electrode was placed in the region of the distal insertion of the sartorius muscle, that is, in the medial part of the thigh, as shown in Figure 2. Rectangular pulses with a duration of 0.2 ms, a frequency of 1 Hz, and an intensity in the range of 0-20 mA were applied and adjusted to obtain a clear sensory nerve action potential (SNAP) [20]. Signal recording was performed using an active electrode placed medially to the border of the tibia, approximately 10-12 cm above the medial malleolus, while the reference electrode was positioned 2-3 cm distally. The ground electrode was placed on the lower leg.

The following parameters were evaluated: SNAP amplitude in µV, sensory latency in ms, and sensory conduction velocity (SCV) in m/s, calculated based on the known distance between the stimulating and recording electrodes [21].

Statistical analysis included bilateral side-to-side comparisons, Spearman rank correlation analysis, and Pearson correlation analysis, according to data type and distribution. The normality of distribution was assessed using the Shapiro-Wilk test prior to the selection of the correlation method. A p-value of less than 0.05 was considered statistically significant. Statistical analysis was performed using JASP version 0.96 (University of Amsterdam, Amsterdam, Netherlands).

## Results

In the analysis of femoral nerve motor conduction, high symmetry between the limbs was observed in terms of both latency and CMAP amplitude. The comparison of the values obtained for the left and right limbs did not reveal statistically significant differences (p > 0.05), which confirms the stability of motor conduction parameters in a healthy population. For the sensory conduction of the saphenous nerve, high symmetry between the limbs was also observed (p > 0.05).

The mean sensory latency of the saphenous nerve was 3.33 ± 1.06 ms, while the mean SNAP amplitude was 3.79 ± 2.10 µV. The mean sensory conduction velocity was 53.75 ± 12.78 m/s (Table 2).

**Table 2:** Saphenous sensory nerve action potential (SNAP) parameters (n = 30)

Parameter	Mean	SD
Latency (ms)	3.33	1.06
SNAP amplitude (µV)	3.79	2.1
Conduction velocity (m/s)	53.75	12.78

In the motor conduction study of the femoral nerve, the mean distal latency was 3.46 ± 1.10 ms, the CMAP amplitude was 10.57 ± 3.37 mV, and the conduction velocity was 69.67 ± 11.70 m/s (Table 3).

**Table 3:** Femoral compound muscle action potential (CMAP) parameters (n = 30)

Parameter	Mean	SD
Latency (ms)	3.46	1.1
CMAP amplitude (mV)	10.57	3.37
Conduction velocity (m/s)	69.67	11.7

The flexibility of the posterior thigh tissues, assessed using the popliteal angle, showed a moderate positive correlation with SNAP amplitude during the stimulation of the saphenous nerve at the distal insertion of the sartorius muscle (ρ = 0.55; p < 0.05; Spearman). This result indicates that greater soft-tissue flexibility is associated with a greater sensory response.

The age of the participants correlated positively with the latency of the saphenous nerve during stimulation at the distal insertion of the sartorius muscle (ρ = 0.38; p < 0.05; Spearman). Moreover, a negative correlation was observed between age and SNAP amplitude during the same examination (ρ = -0.36; Spearman), which may indicate subtle changes in the function of sensory fibers even in a young adult population.

**Figure 3:**
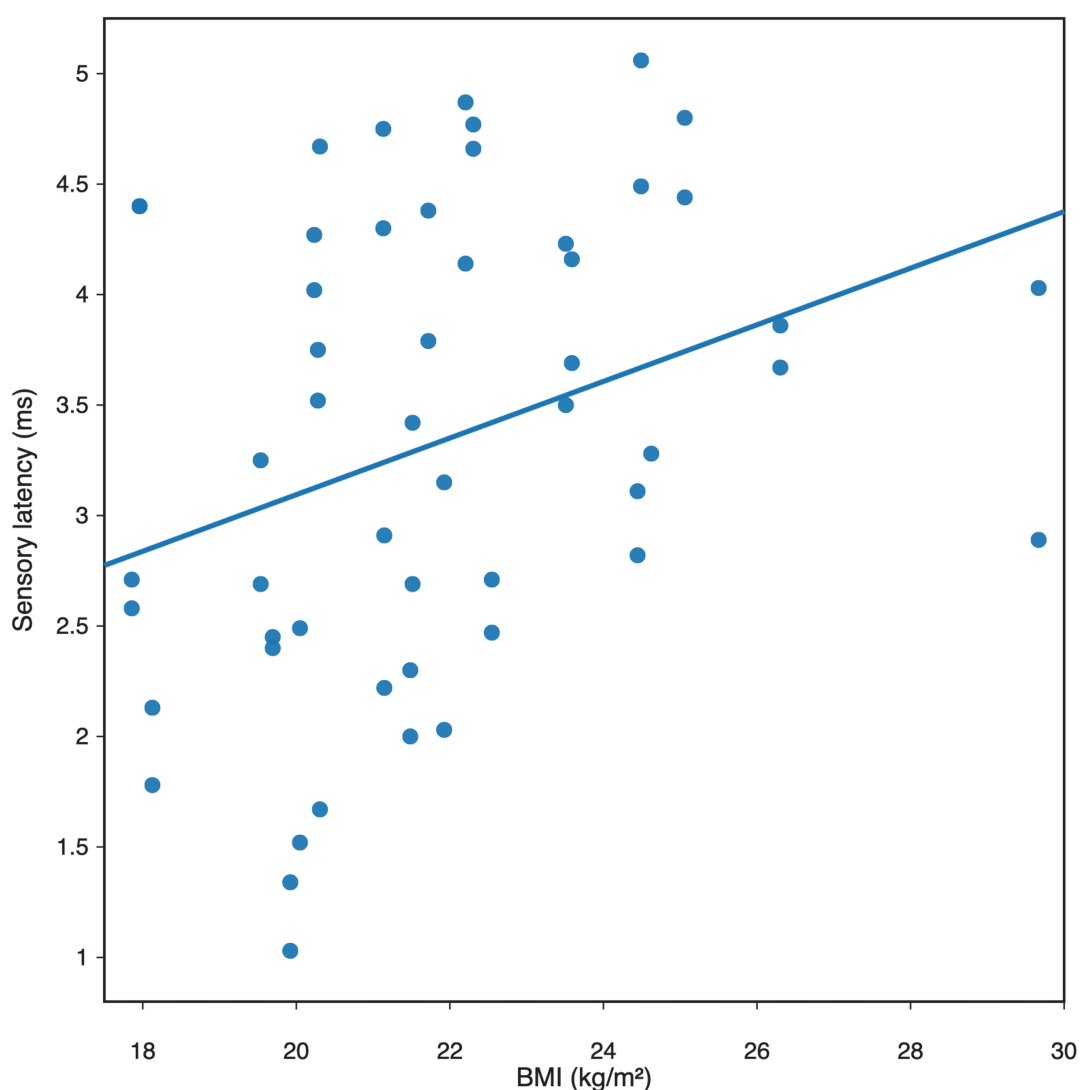
Relationship between sensory latency and BMI in the study population (n = 30 participants) Pearson correlation between BMI and sensory latency: r = 0.32 and p = 0.017 BMI: body mass index

**Figure 4:**
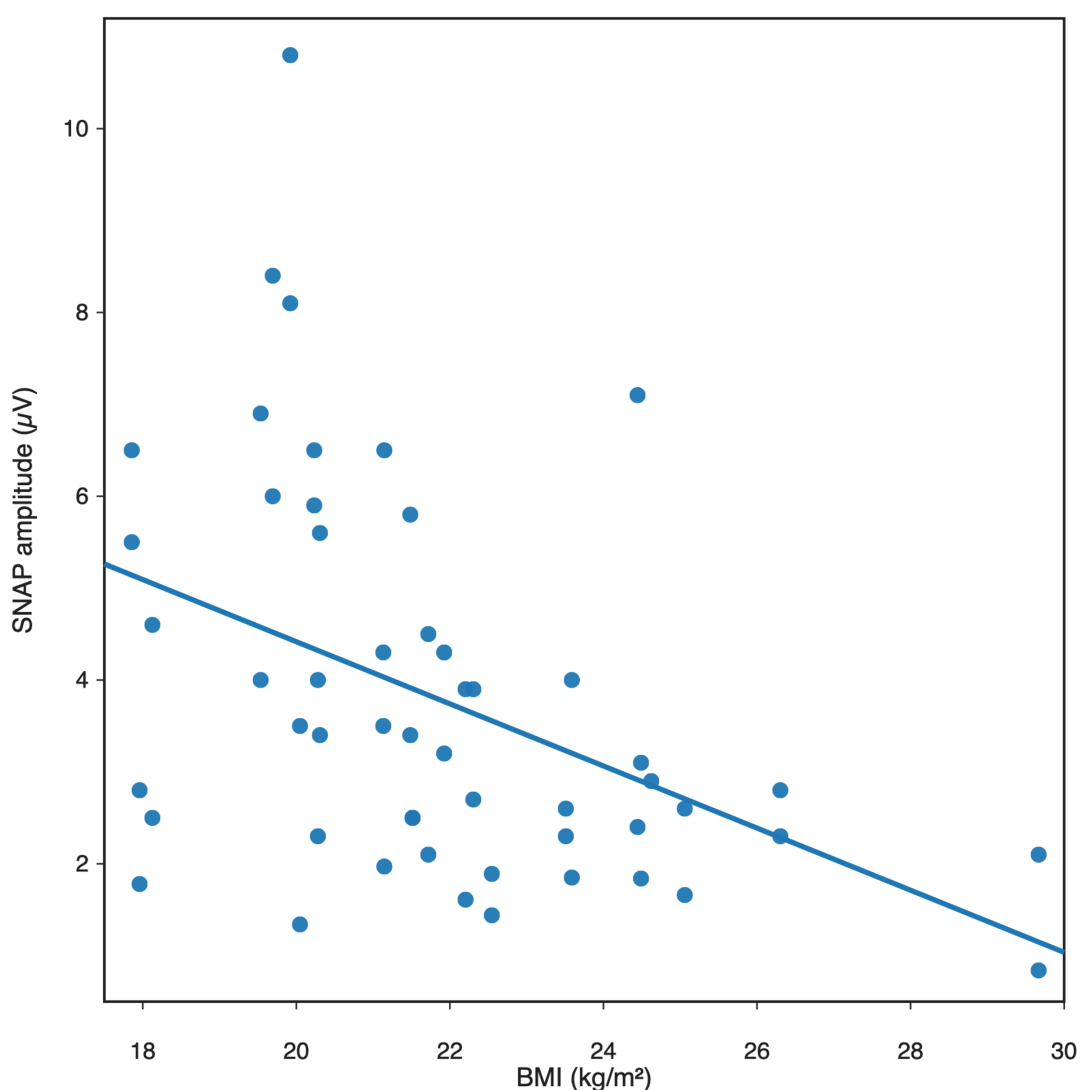
Relationship between saphenous nerve SNAP amplitude and BMI in the study population (n = 30 participants) Pearson correlation between BMI and SNAP amplitude: r = -0.43 and p = 0.001 BMI, body mass index; SNAP, sensory nerve action potential

In the analysis of relationships with anthropometric parameters, BMI showed a negative correlation with sensory conduction parameters of the saphenous nerve recorded during stimulation at the distal insertion of the sartorius muscle. BMI correlated negatively with SNAP amplitude (r = -0.43; Pearson) and positively with sensory latency (r = 0.32; Pearson). This suggests that greater body mass may influence the quality of sensory response recording in the distal segment of the nerve. The relationship between BMI and sensory latency is shown in Figure 3, whereas the relationship between BMI and SNAP amplitude is shown in Figure 4.

## Discussion

The analysis of femoral and saphenous nerve conduction in a healthy population allows the assessment of the natural variability of NCS parameters and provides a reference point for further clinical studies, particularly in the context of metabolic neuropathies involving the femoral nerve [22]. The obtained results confirmed a very high symmetry of femoral nerve conduction, which is consistent with the literature concerning large motor nerves characterized by stable morphology and low susceptibility to subclinical changes [23]. It is worth emphasizing that the CMAP amplitude of the femoral nerve demonstrates relatively low interindividual variability in a population of young healthy individuals, as confirmed by the results of the present study [24]. With regard to the saphenous nerve, strong bilateral symmetry, particularly at the lower leg recording site, confirms that even superficially located sensory nerves may demonstrate high functional stability in healthy individuals [25]. The mean SNAP amplitude and latency values obtained in the present study are consistent with previously published normative data for antidromic saphenous nerve conduction [26]. One of the key findings was the positive correlation between lower-limb flexibility and SNAP amplitude. Similar observations regarding the influence of physical activity on lower-limb conduction parameters were reported by Colak et al. [27]. This phenomenon may be explained by the improved gliding of the saphenous nerve within the surrounding soft tissues when hamstring flexibility is greater. Reduced flexibility could increase tension within fascial structures and create mechanical constraints affecting nerve conduction [28]. In the present cohort, BMI was associated only with sensory parameters of the saphenous nerve and was not related to femoral motor conduction. This pattern is compatible with reports indicating that the influence of body habitus is more evident in sensory recordings, where subcutaneous tissue thickness can affect signal acquisition [29,30]. The relationship between sensory latency and age may reflect early subclinical changes that occur even in young adults [23]. Although these changes are subtle, they may influence sensory conduction parameters. Overall, the results of the present study indicate that the flexibility of lower-limb soft tissues may be a factor associated with saphenous nerve conduction, which is important for the interpretation of NCS results in clinical practice.

This study has several limitations. First, the sample size was relatively small and consisted exclusively of young, physically active adults, which limits the generalizability of the findings to older individuals, sedentary populations, and patients with clinically evident neuropathy. Second, the cohort comprised 19 women and 11 men; this sex imbalance may have introduced confounding, and sex-related differences in anthropometric variables and nerve conduction parameters cannot be fully excluded in the absence of sex-stratified analyses. Third, age and BMI were analyzed as independent predictors, and their interaction and potential collinearity were not formally assessed, which should be considered when interpreting the independence of the reported correlations. Fourth, the cross-sectional design does not allow causal inference between anthropometric or biomechanical factors and nerve conduction parameters. Fifth, the study was conducted at a single center, and the use of surface recordings may have been influenced by technical and anatomical variability, including interindividual differences in limb length, soft-tissue thickness, electrode-related signal acquisition conditions, and measurement-related artefacts. Finally, although the findings provide clinically relevant normative observations, they should be interpreted as preliminary and confirmed in larger studies with broader demographic representation.

## Conclusions

The analysis of femoral and saphenous nerve conduction in healthy, physically active young adults suggests that neurophysiological parameters tend to exhibit bilateral symmetry, with no meaningful side-to-side differences in latency or response amplitude for either motor or sensory responses. Given the cross-sectional design and limited sample size, these preliminary observations should be interpreted with caution, and larger longitudinal studies are needed before side-to-side comparisons can be recommended as a reference standard in the clinical assessment of neuropathies.

The greater flexibility of the hamstring muscle group, as measured by the popliteal angle, was associated with enhanced sensory nerve responses during stimulation near the distal insertion of the sartorius muscle. This suggests that the mechanical properties of surrounding tissues may influence local nerve conduction. Additionally, relationships between age, body mass index, and sensory nerve conduction parameters indicate that subtle variations in afferent nerve function may occur even in a young adult population. Increasing age was associated with a tendency toward slower sensory responses, while higher body mass index was linked to reduced response amplitude and prolonged latency. Although these variations are not clinically significant in asymptomatic individuals, they should be taken into account when interpreting neurophysiological findings, particularly in sensory recordings of the saphenous nerve.
